# Development of an Adult Daycare Center Service Model for the Elderly Through Community Participation: An Action Research Approach

**DOI:** 10.3390/geriatrics10020055

**Published:** 2025-04-04

**Authors:** Benjayamas Pilayon, Kanin Chueaduangpui, Juthaluck Saentho, Ruchakron Kongmant, Niruwan Turnbull

**Affiliations:** 1 College of Nursing Nakhon Phanom, Nakhon Phanom University, Nakhon Phanom 48000, Thailand; benjayamas@npu.ac.th; 2 Faculty of Liberal of Arts and science, Nakhon Phanom University, Nakhon Phanom 48000, Thailand; kanincd2@gmail.com (K.C.); juthalucks@npu.ac.th (J.S.); 3 Public Health and Environmental Policy in Southeast Asia Research Cluster (PHEP-SEA), Mahasarakham University, Maha Sarakham 44150, Thailand; ruchakron.k@msu.ac.th; 4 Faculty of Public Health, Mahasarakham University, Maha Sarakham 44150, Thailand

**Keywords:** daycare center, elderly, community participation, participatory action research, service model, Nakhon Phanom

## Abstract

**Introduction**: This study aimed to develop a service model for daycare centers for the elderly through community participation using participatory action research methods. The objectives were threefold: (1) to investigate the current situation of the elderly in the community and their needs for daycare center services, (2) to develop a daycare center for the elderly with active community involvement, and (3) to evaluate the effectiveness of the service delivery at the daycare center for the elderly. **Methods**: The study was conducted in Ban Kho Subdistrict, Phon Sawan District, Nakhon Phanom Province. Research participants included 210 elderly individuals surveyed to assess their situation, and 15 key informants, including elderly club leaders, subdistrict health promotion hospital staff, volunteers, subdistrict administrative organization officers, and village health volunteers, were specifically selected for in-depth insights. The research process was structured into three phases: Phase 1 focused on studying the situation of the elderly in the community and their service needs; Phase 2 was dedicated to developing the daycare center with community participation; and Phase 3 involved evaluating the service delivery of the daycare center. **Results:** The results indicated that the development process of the daycare center service model for the elderly, through community participation, involved four key mechanisms: elderly clubs, subdistrict health promotion hospitals, volunteer teachers or technicians, and village volunteers. Additionally, the supporting mechanisms included academic institutions, hospitals, temples, village heads, the Non-Formal Education Center, foundations, and the subdistrict administrative organization. The comprehensive service model encompassed five components: health, social, psychological, economic, and environmental aspects. **Conclusions**: The study successfully developed a daycare center service model for the elderly through community participation, which can be expanded and adapted to other semi-urban and semi-rural contexts. This model demonstrates the importance of community involvement in providing holistic care for the elderly, addressing various aspects of their well-being.

## 1. Introduction

The aging population is a global phenomenon, with significant implications for social and healthcare systems [1]. In Asia, this demographic shift is particularly pronounced. Asia is home to 60% of the world’s elderly population, with countries such as Japan, South Korea, China, and Thailand experiencing rapid increases in the number of elderly individuals [2]. This demographic trend presents both opportunities and challenges for policymakers and service providers across the region. Thailand, in particular, is witnessing a dramatic demographic transformation. According to the National Statistical Office of Thailand, the number of elderly individuals (defined as those aged 60 and above) is expected to rise from 13% of the total population in 2010 to 30% by 2050 [3]. This increase in the elderly population is accompanied by a decline in the working-age population, leading to potential economic and social challenges [4]. These demographic shifts necessitate a re-evaluation of existing social and healthcare infrastructures to support the growing elderly population. The traditional family-based care system in Thailand is under pressure due to urbanization, migration, and changes in family structure. As younger family members move to urban areas for better economic opportunities, the elderly are often left in rural areas with limited access to adequate care and support services [5]. This shift necessitates the development of formal care services, such as daycare centers for the elderly, to fill the gap left by the weakening family support system.

Thailand’s healthcare system has evolved to address these challenges by establishing a robust Universal Coverage Scheme (UCS), providing healthcare access to nearly all citizens. Managed by the National Health Security Office (NHSO), the UCS is predominantly funded through general taxation and offers comprehensive medical services, including primary care, hospitalizations, and preventive healthcare [1]. Complementary health coverage is provided by the Social Security Scheme (SSS), funded by contributions from employees, employers, and the government, as well as the Civil Servant Medical Benefit Scheme (CSMBS), covering government employees and their families [2]. Despite the strong emphasis on family-based caregiving in Thai culture, recent socioeconomic trends—such as urbanization, migration, and changes in family structures—have strained traditional informal support systems [3]. Younger generations increasingly migrate to urban areas seeking economic opportunities, leaving elderly individuals, especially in rural areas, with reduced familial support and limited access to adequate care and social interaction. This changing family dynamic has necessitated the development of formal care services, including community-based elderly daycare centers, to bridge gaps in elderly care [4]. Social care for the elderly in Thailand falls under the Department of Older Persons (DOP) within the Ministry of Social Development and Human Security (MSDHS), which provides welfare assistance and supports community-driven initiatives like elderly daycare centers. The government’s commitment includes financial measures such as the Old-Age Allowance Scheme, providing monthly stipends to elderly individuals to enhance their economic security [5].

Nakhon Phanom Province, located in a special economic zone, exemplifies these demographic trends. A survey conducted by the Tha Kha Subdistrict Health Promoting Hospital (Ban Kho Subdistrict Health Promoting Hospital) in Ban Kho Subdistrict, Phon Sawan District, Nakhon Phanom Province, revealed that the elderly population is categorized as follows: 227 individuals aged 60–69 years, 110 individuals aged 70–79 years, 14 individuals aged 80–84 years, and 17 individuals aged 85 years and above. In total, there are 363 elderly individuals, accounting for 10.46% of the total population. Ban Kho Subdistrict is transitioning into an aging society and will soon become a fully aged society. Among the elderly, 160 are males (44.07%) and 203 are females (55.93%) [3].

The health data from the survey indicate that 40 elderly individuals (12.39%) suffer from hypertension, 45 individuals (12.39%) have diabetes, 4 individuals (1.10%) suffer from other chronic diseases such as asthma, chronic obstructive pulmonary disease (COPD), and heart disease, and 2 individuals (0.55%) have cerebrovascular disease. Additionally, there are 8 bedridden elderly individuals (2.20%), 42 home-bound elderly individuals (11.57%), and 314 socially active elderly individuals (86.77%). Furthermore, eight elderly individuals live alone (2.20%) [6]. The prevalence of chronic illnesses and the high percentage of elderly living alone highlight the urgent need for comprehensive elderly care services.

Community participation is a crucial element in the successful implementation and sustainability of elderly care services [7]. By involving community members in the development and operation of daycare centers, services can be tailored to meet the specific needs of the elderly population within a particular locality, ensuring a higher level of acceptance and effectiveness [8]. Participatory action research (PAR) is an effective methodology for engaging communities in the research process, fostering collaboration, and generating practical solutions that address local challenges [9]. PAR emphasizes collaborative problem-solving and the empowerment of community members, which can lead to more sustainable and impactful interventions.

This study aimed to develop a service model for daycare centers for the elderly through community participation, employing PAR methods to achieve the following objectives: (1) to investigate the current situation of the elderly in the community and their needs for daycare center services, (2) to develop a daycare center for the elderly with active community involvement, and (3) to evaluate the effectiveness of the service delivery at the daycare center. The research was conducted in Ban Kho Subdistrict, Phon Sawan District, Nakhon Phanom Province, an area characterized by a semi-urban and semi-rural context. This region, with its unique blend of urban and rural characteristics, provides an ideal setting for developing and testing community-driven care models.

The findings from this study are expected to provide a comprehensive service model that can be adapted and implemented in similar settings, ultimately enhancing the well-being of elderly individuals through community-driven care initiatives. By integrating community participation into the development and implementation of elderly care services, the study aims to create a model that is not only effective but also sustainable and culturally relevant. This paper will outline the research methods, present the results, and discuss the implications of the developed service model for future elderly care practices.

## 2. Methods

**Figure 1:**
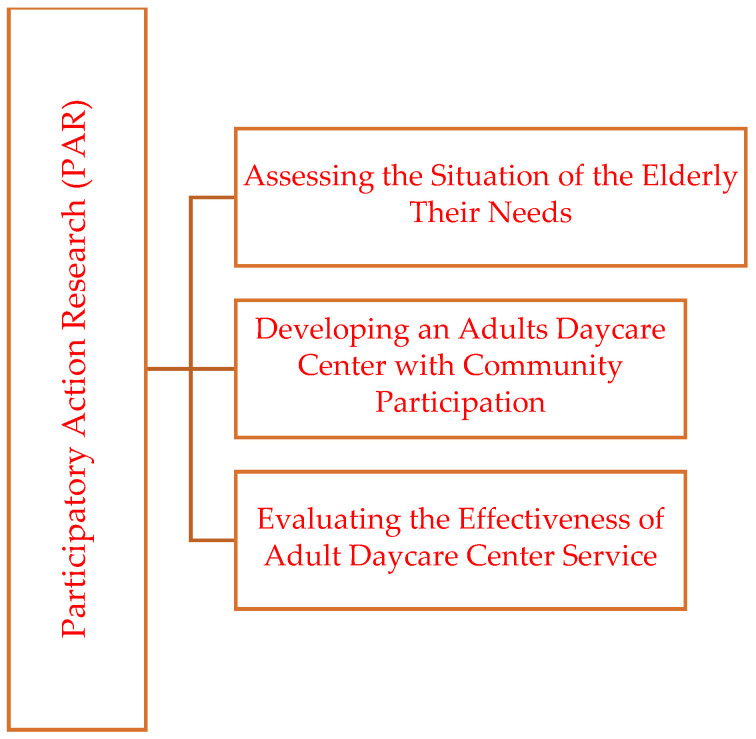
The Participatory Action Research (PAR) Phases.

This study employed Participatory Action Research (PAR) methodologies, structured into three phases: (1) assessing the situation of the elderly in the community and their needs for daycare center services, (2) developing a daycare center for the elderly with community participation, and (3) evaluating the effectiveness of the daycare center services for the elderly (Figure 1).

### 2.1. Population and Sample

The study focused on the elderly population in Ban Kho Subdistrict, Phon Sawan District, Nakhon Phanom Province. The sample size was determined using Yamane’s [10] formula with a margin of error of ±10% for a population of 1500, resulting in a minimum required sample size of 94. The decision to use a 10% margin of error was based on practical considerations, including logistical and resource constraints, while ensuring a sufficiently representative sample. To enhance the robustness of the findings, the sample size was expanded to 210 participants, exceeding the minimum requirement and aligning with Hair’s [11] recommendation for larger sample sizes in community-based studies.

To ensure a representative sample, a two-stage random sampling method was applied:

(1) Cluster Sampling: the subdistrict was divided into 20 villages to capture geographic and demographic diversity.
(2) Simple Random Sampling: within each village, elderly individuals were randomly selected using a computerized randomization process to minimize selection bias.

The representativeness of the sample was evaluated based on demographic characteristics, including age distribution, socioeconomic status, and chronic health conditions, ensuring it adequately reflected the elderly population in the study area.

To focus on elderly individuals who could actively engage in daycare services, the study applied the following inclusion and exclusion criteria:

#### 2.1.1. Inclusion Criteria

- Elderly individuals aged 60 years and above residing in Ban Kho Subdistrict, Phon Sawan District, Nakhon Phanom Province.
- Permanent residents of the community.
- Individuals with chronic illnesses who are ambulatory and able to participate in activities (e.g., diabetes, hypertension) as identified in the community health survey.
- Socially active elderly individuals.
- Participants who provided informed consent.
- Key community informants, such as elderly club leaders, subdistrict health promotion hospital staff, and village health volunteers.

#### 2.1.2. Exclusion Criteria

- Bedridden or homebound elderly individuals unable to participate in community activities.
- Individuals with severe cognitive impairments (e.g., advanced dementia) that limit engagement in study activities.
- Temporary residents or individuals living outside the study area.
- Participants who declined to give consent or withdrew from the study.
- Individuals facing logistical barriers, such as lack of transportation or caregiver support.

#### 2.1.3. Functional Status Assessment

Participants’ functionality was assessed using the Barthel Activities of Daily Living (ADL) Index, a widely used tool for evaluating an individual’s ability to perform essential daily tasks independently. This ensured that selected participants could actively participate in the daycare center’s activities.

### 2.2. Implementation Phases and Quality Control

The development of the daycare center was structured into three key phases.

#### 2.2.1. Phase 1: Planning and Community Engagement

Initial consultations were held with community stakeholders, including local health workers, elderly club representatives, and village health volunteers. The needs assessments were conducted to design a service model tailored to the specific needs of the elderly population.

#### 2.2.2. Phase 2: Service Implementation

A standardized framework for daycare activities was developed, including health screenings, nutritional programs, cognitive stimulation exercises, and social engagement activities. Care providers and volunteers received structured training on elderly care protocols, supervised by healthcare professionals. The daycare center operated under strict adherence to national elderly care standards, with services delivered in collaboration with the local health promotion hospital.

#### 2.2.3. Phase 3: Monitoring and Evaluation

A structured monitoring system was implemented to assess the effectiveness of services through regular observations and participant feedback. Feedback mechanisms included bi-weekly focus group discussions with elderly participants and caregivers to refine the intervention model. Data on health outcomes, social participation, and quality of life indicators were systematically collected and analyzed to ensure service effectiveness and sustainability.

### 2.3. Research Tools

The primary data collection tool for this study was a semi-structured interview, which underwent rigorous validation for content accuracy, language appropriateness, and comprehensiveness by a panel of three experts. Based on their feedback, necessary adjustments were made to enhance the tool’s effectiveness. To further refine the interview tool, pilot interviews were conducted with individuals who had characteristics similar to the actual study participants.

### 2.4. Data Collection and Reliability Concern

The combination of these rigorous methods for data collection, reliability, and validity assessments ensured that the study’s findings were robust, trustworthy, and applicable to broader contexts. The data collection process followed the following steps:

(1) Preparation and Validation: The semi-structured interview tool was developed and reviewed by three experts. Feedback was incorporated to ensure content accuracy and appropriateness.
(2) Pilot Testing: pilot interviews were conducted with individuals resembling the study participants to refine the tool and ensure its effectiveness.
(3) Training: The research team received extensive training in qualitative research methodologies and had substantial experience in conducting qualitative studies. This training ensured that the data collected would be reliable and valid.

Data reliability and validity were assessed using the criteria proposed by Lincoln and Guba [12], which are widely recognized in qualitative research. These criteria included the following:

(1) Credibility: ensuring the data accurately reflected participants’ experiences by involving those with direct experience of the phenomena under investigation.
(2) Transferability: providing rich, detailed descriptions of the findings to enable other researchers to determine the applicability of the results to similar contexts.
(3) Dependability: meticulously documenting the research process to ensure consistent procedures and agreement among the research team.
(4) Conformability: using activity logs and reflective notes to allow verification by other researchers and employing triangulation techniques, which involved cross-checking information from multiple sources and methods, with data reviewed by two independent experts.

### 2.5. Data Analysis

**Figure 2:**
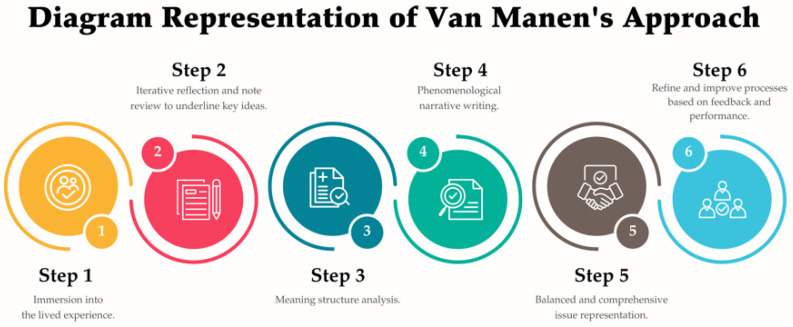
Diagram illustrating Van Mane’s approach to data analysis.

The data analysis in this study followed the Van Manen [13] approach, consisting of six steps: (1) understanding the nature of the lived experience, (2) exploring the studied experiences through repeated reflection on the data and reviewing field notes while underlining key words and phrases, (3) interpreting the data by reflecting on essential meaning structures, (4) writing a phenomenological description and selecting sentences or meaning units to present, (5) checking the consistency of the studied phenomenon, and (6) ensuring the balance of the presented issues (Figure 2). The data analysis in this study employed both descriptive and inferential statistical methods to evaluate the effectiveness of the daycare center intervention.

#### 2.5.1. Data Description

Demographic and clinical data of participants were summarized using descriptive statistics, including means, standard deviations, and frequency distributions. Confidence intervals (95% CI) for key estimates, such as weight reduction, BMI changes, and functional improvements, were calculated and reported to enhance statistical interpretation. Normality tests (Kolmogorov–Smirnov and Shapiro–Wilk tests) were conducted to determine whether data followed a normal distribution before applying parametric tests. All variables met the assumptions for normality, justifying the use of parametric statistical methods.

#### 2.5.2. Comparative Analysis

A paired *t*-test was used to compare pre- and post-intervention differences within the same participants, ensuring that the independence assumption of samples was appropriately addressed. Baseline comparability was verified using independent t-tests and chi-square tests to confirm that there were no significant pre-intervention differences in key demographic or health variables. The reported weight reduction (0.64 kg) and BMI decrease (0.28) were statistically significant (*p* < 0.05). However, to assess clinical relevance, the results were compared with established guidelines on meaningful weight changes in elderly populations. The observed reductions, though modest, align with recommendations for gradual weight loss to improve metabolic health in older adults. Functional improvements according to the Barthel Index were measured immediately after the intervention and at a 3-month follow-up. The results demonstrated sustained improvement in functional ability over time, confirming the long-term impact of the daycare intervention.

#### 2.5.3. Instrument Reliability

The semi-structured questionnaire and interview tools underwent expert validation, and reliability was assessed using Cronbach’s alpha, which yielded a value of 0.85, indicating high internal consistency. The dementia assessment was conducted using the Thai Mental State Examination (TMSE), a validated cognitive screening tool for elderly populations. This ensures replicability and enhances the study’s methodological rigor.

Additionally, we used SPSS Version 20.0 to analyze the quantitative data related to general information, ensuring precise and reliable statistical computations. As part of the analysis, frequencies were used to find out how categorical variables were distributed, percentages were used to show how many responses there were for each category, and standard deviations were used to see where the continuous variables were. We systematically entered the data into SPSS, clearly defining and coding the variables for efficient processing. We generated descriptive statistics through SPSS to gain a comprehensive understanding of the findings, which included detailed insights into demographic characteristics, health status, and socioeconomic conditions. We further utilized these outputs to summarize and present the data in a clear, interpretable format, ensuring accuracy and alignment with the research objectives.

### 2.6. Project Implementation Financial Considerations

#### 2.6.1. Establishment of the Daycare Center

The development of the daycare center for the elderly in Ban Kho Subdistrict followed a community-driven approach involving local administrative organizations, healthcare institutions, and volunteer groups. The project was structured in three key phases:

(1) *Planning and Community Engagement*: Initial consultations were conducted with stakeholders, including village health volunteers, local administrative officers, elderly club representatives, and healthcare workers. These discussions helped identify the needs of the elderly population and secure community support.
(2) *Infrastructure Setup:* The project leveraged existing community facilities to minimize construction costs. A multipurpose community building was renovated to accommodate elderly-friendly services, including designated areas for social interaction, recreational activities, and basic healthcare services.
(3) *Service Design and Implementation:* The daycare center was designed to operate on weekdays, providing health screenings, social engagement programs, cognitive stimulation activities, and nutritional support for elderly participants.

#### 2.6.2. Financial Considerations and Sustainability

The implementation and operation of the daycare center required both initial investment and ongoing operational funding, sourced from a mix of local government budgets, community fundraising, and in-kind contributions from stakeholders.

**A. Cost Breakdown**

The estimated costs of setting up and running the daycare center are summarized below (See Table 1).

**B. Funding Sources and Cost-Effectiveness**

The sustainability of the daycare center is ensured through a diversified funding approach, reducing reliance on any single entity.

1. *Government Contributions:*

The Subdistrict Administrative Organization (SAO) allocates an annual budget for operational costs. Public Health Office partnerships provide free medical check-ups and healthcare staff.

2. *Community Fundraising and Private Donations:*

Temple-led charity drives, local business sponsorships, and household donations contribute to recurring expenses. Non-governmental organizations (NGOs) provide one-time grants for equipment and training programs.

3. *Academic and Institutional Partnerships:*

Universities co-fund research and training activities that align with elderly care. Students and faculty from Mahasarakham University and Nakhon Phanom University contribute through volunteer work and capacity-building initiatives.

### 2.7. Ethical Considerations

The study received ethical approval from the Human Research Ethics Committee (approval number: 26/64Exp.). The research process adhered to ethical guidelines throughout the data collection phase, ensuring the rights and confidentiality of all participants were protected.

## 3. Results

### 3.1. Phase 1: Investigated the Current Situation and Specific Needs of the Elderly

#### 3.1.1. The Socioeconomic Information and the Specific Needs of the Elderly

This phase investigated the current situation and specific needs of the elderly in the community, covering four key areas: health, economic, social, and environmental problems and needs. It provided a comprehensive understanding of the challenges faced by the elderly and their requirements for improving their quality of life. The study found that most elderly individuals were female, with an average age of 69.17 years, and a significant portion suffered from chronic diseases. The majority were engaged in agriculture and had low income levels, relying on allowances that were often insufficient. The health needs were found to be high, with a substantial demand for regular check-ups and professional care. Additionally, a strong demand for establishing a daycare center for the elderly, with the need for daycare center establishment scoring an average of 4.47, service needs at 4.33, and facility needs at 4.20, all interpreted as high. The overall score for the questionnaire on daycare service needs was 4.33, further emphasizing the significant demand for such services and facilities (See Table 2, Table 3, Table 4 and Table 5).

#### 3.1.2. The Participants Approaching

**Figure 3:**
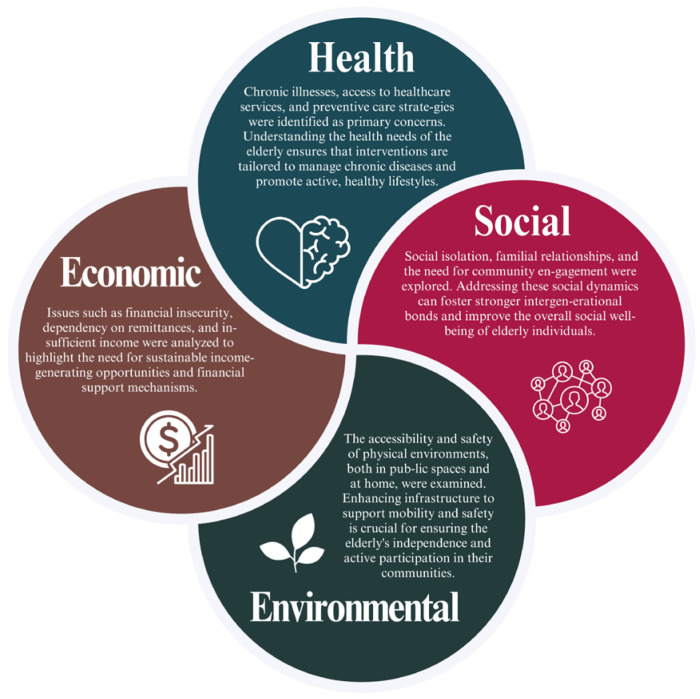
The diagram illustrates the correlation between four key areas, namely health, economic, social, and environmental, that are related to elderly life.

By focusing on these areas, we employed 20 elderly people to join our research. The research provides a holistic view of the elderly’s lives, allowing policymakers, healthcare providers, and community planners to design effective and targeted programs that address the comprehensive needs of the elderly population. The study emphasized four key areas critical to understanding the challenges faced by elderly individuals: **health, economic, social**, and **environmental** aspects (See Figure 3).

- *Health:* Chronic illnesses, access to healthcare services, and preventive care strategies were identified as primary concerns. Understanding the health needs of the elderly ensures that interventions are tailored to manage chronic diseases and promote active, healthy lifestyles.
- *Economic*: issues such as financial insecurity, dependency on remittances, and insufficient income were analyzed to highlight the need for sustainable income-generating opportunities and financial support mechanisms.
- *Social*: Social isolation, familial relationships, and the need for community engagement were explored. Addressing these social dynamics can foster stronger intergenerational bonds and improve the overall social well-being of elderly individuals.
- *Environmental*: The accessibility and safety of physical environments, both in public spaces and at home, were examined. Enhancing infrastructure to support mobility and safety is crucial for ensuring the elderly’s independence and active participation in their communities.

By systematically addressing these areas, the research facilitates the development of effective, multidimensional interventions and support systems that align with the specific needs of the elderly population, fostering their health, independence, and quality of life.

***I. Health Challenges and Needs among the Elderly***

*(1) Health Issues:* A significant proportion of elderly individuals in the study suffer from chronic health conditions such as diabetes and hypertension. These conditions are generally managed through regular medication provided by local health promotion hospitals. Despite these health challenges, many elderly individuals remain actively engaged in daily activities, including farming and animal husbandry. For instance, Mr. Tawee, a 72-year-old participant, shared, “*I take medication for hypertension and diabetes regularly and follow the doctor’s instructions. By eating healthy, I manage to stay well and not burden others*”.

*(2) Concerns:* A prominent concern among the elderly is the potential progression and worsening of their chronic diseases as they age. This concern underscores the need for comprehensive support systems, including regular health monitoring and guidance on managing chronic conditions. The elderly have expressed a desire for increased home visits by healthcare volunteers, tailored advice on self-care, and opportunities to share experiences and knowledge with peers. As Mrs. Mee, a 76-year-old participant, expressed, “*As I age, I worry about my chronic conditions worsening. I hope for more frequent home visits from healthcare professionals to monitor my health and provide self-care advice*”.

***II. Economic Challenges and Needs among the Elderly***

The majority of elderly individuals in the study possess land for agricultural purposes and engage in seasonal farming activities. Some supplement their income through handicraft production. However, the primary sources of income for most participants include remittances from their children, who often work in other regions, and government-provided elderly allowances. Despite these income sources, many elderly individuals face economic hardships, including debts incurred from agricultural loans and the financial burden of caring for grandchildren. For example, Mrs. Pien, a 70-year-old participant, noted, “*I work on the farm and manage household expenses with support from my children and the elderly allowance*”.

To address these challenges, elderly individuals require support to develop self-sustaining occupations and improve their financial stability. Interventions such as targeted assistance for impoverished elderly individuals, the establishment of community-based elderly aid funds, and the promotion of financial savings through local welfare mechanisms are crucial. Additionally, there is a need for initiatives aimed at enhancing income-generating activities, such as improving the production and marketability of handicrafts. As expressed by Mrs. Mee, a 76-year-old participant, “*I wish for assistance in improving our handicraft work to increase income and recognition, reducing our dependency on children*”.

***III. Social Challenges and Needs among the Elderly***

*(1) Social Issues:* The community is characterized by strong kinship ties and mutual support, which provide a foundation for collective well-being. However, several social challenges persist, including issues such as drug abuse and juvenile delinquency. For elderly individuals, these challenges are compounded by strained familial relationships and caregiving responsibilities, which can limit their ability to actively participate in community activities. These barriers not only affect their social engagement but may also contribute to feelings of isolation and stress.

*(2) Social Needs:* To address these challenges, there is a pressing need for relevant agencies and organizations to provide targeted support to elderly caregivers. Initiatives aimed at promoting intergenerational harmony and understanding within families are particularly crucial. Additionally, elderly individuals have expressed a strong desire for more community activities that encourage social engagement, strengthen relationships, and foster a sense of belonging.

For instance, Mr. Kaew, a 72-year-old participant, noted, “*I hope for support in addressing my granddaughter’s behavior and facilitating community activities for the elderly*”. His perspective underscores the need for holistic approaches that integrate family dynamics with community engagement to enhance the social well-being of elderly individuals.

***IV. Environmental Challenges and Needs among the Elderly***

*(1) Community Environment*: The rural environment provides limited public facilities specifically designed to accommodate the needs of elderly individuals. While the local health promotion hospital is well-equipped and accessible, other commonly used spaces, such as temples, present significant mobility challenges due to inadequate infrastructure. These barriers restrict the elderly from fully participating in social and religious activities, which are vital for their overall well-being. As Mr. Tawee, a 72-year-old participant, explained, “*The health promotion hospital is well-equipped, but other places like temples need better facilities for the elderly*”.

*(2) Environmental Needs:* To improve the quality of life for the elderly, there is a pressing need for enhanced public spaces equipped with accessible facilities. This includes installing ramps, grab bars, and other structural modifications to ensure safety and ease of use. Additionally, regular assessments of home environments are necessary to identify and address safety concerns, promoting independent living among elderly residents. Highlighting this need, Mr. Som, a 76-year-old participant, shared, “*We need funding to improve common areas and ensure safe facilities like ramps and grab bars in public restrooms*”.

#### 3.1.3. Role of Community Leaders in Assisting the Elderly

The involvement of community leaders plays a pivotal role in recognizing and mobilizing existing support structures while addressing the unique challenges faced by the elderly population. This leadership is instrumental in evaluating the effectiveness of local governance and its capacity to respond to the multifaceted needs of elderly individuals. By examining their involvement, this study highlights the critical role of community-based interventions and the importance of equipping leaders with the necessary tools, knowledge, and resources to advocate for and implement effective strategies. Community leaders, such as village heads, health volunteers, and social development volunteers, serve as intermediaries between the elderly and various service providers. Their responsibilities often include identifying the needs of the elderly, facilitating access to essential services, and fostering collaboration among stakeholders. These leaders also promote community participation in elderly care programs, ensuring that services are culturally appropriate and aligned with local needs.

For instance, health volunteers conduct assessments of the elderly’s health and social needs, referring them to appropriate services, such as healthcare, psychological support, or economic aid. This proactive approach ensures that the elderly receive timely and comprehensive assistance. As Mrs. Pien, a 70-year-old participant, shared, “*Health volunteers assess the elderly’s needs and refer them to appropriate services, such as healthcare or social support*”. Moreover, community leaders often play a critical role in organizing educational and awareness programs for families and caregivers, emphasizing the importance of intergenerational harmony and effective caregiving practices. They also work to improve accessibility to resources and services by advocating for policy changes or infrastructure improvements, such as transportation to health facilities or enhancements to public spaces.

#### 3.1.4. Role of the Elderly in Community Engagement

Analyzing the engagement of elderly individuals in their communities provides valuable insights into their contributions to social cohesion and cultural preservation. Their active participation not only strengthens communal bonds but also serves as a mechanism for maintaining traditional practices and transferring valuable knowledge to younger generations. This engagement underscores the vital role of the elderly as custodians of cultural heritage and contributors to the well-being of their communities. Elderly individuals actively participate in a range of community activities, primarily facilitated through local elderly associations. These associations organize cultural events, promote traditional practices, and foster intergenerational knowledge sharing. Such activities provide the elderly with opportunities to remain socially connected and contribute to their communities in meaningful ways, ultimately enhancing their mental and social well-being while reducing feelings of isolation.

For example, Mrs. Jumpee, a 64-year-old participant, emphasized, “*The elderly are essential in cultural practices and knowledge transfer within the community*”. This statement highlights the significant role of elderly individuals in sustaining traditions and enriching the community’s cultural fabric. Moreover, the elderly often serve as mentors and advisors within their communities, offering guidance on issues ranging from agricultural practices to family relationships. Their involvement in decision-making processes further demonstrates their capacity to contribute to community development. Participation in these activities not only provides the elderly with a sense of purpose and belonging but also fosters a reciprocal relationship where their wisdom and experience are valued.

#### 3.1.5. Services Required in a Daycare Center for the Elderly

Identifying and addressing the specific services required in a daycare center for the elderly is essential to ensure these facilities effectively cater to their diverse needs. A well-designed daycare center not only supports the physical, social, and mental well-being of elderly individuals but also encourages their active participation in community life. The following services and features are critical to achieving these goals:

(1) *Facility Requirements:* The physical environment of a daycare center should prioritize accessibility and safety to accommodate the mobility challenges often faced by the elderly. Key requirements include the following:
  1. *Accessible Restrooms*: equipped with grab bars and non-slip surfaces to ensure safety and ease of use.
  2. *Open Spaces*: designated areas for communal gatherings and group activities.
  3. *Outdoor Areas*: spaces designed for light exercise, gardening, or simply enjoying nature.

For example, Mr. Som, a 76-year-old participant, suggested, “*The day care center should have facilities similar to the health promotion hospital, with grab bars and accessible restrooms*”.

(2) *Activity Requirements*: Comprehensive programming is essential to address the physical, mental, and social needs of the elderly. Such activities should include the following:
  4. *Health Services*: regular health screenings, exercise sessions, and wellness programs.
  5. *Vocational Training*: opportunities for skill development, such as handicrafts or agricultural techniques that allow the elderly to remain productive and engaged.
  6. *Social Engagement*: group activities, such as music therapy, storytelling sessions, or cultural events, to foster social connections and reduce isolation.

Additionally, retired professionals among the elderly can be involved as instructors or mentors, leveraging their skills and experience to benefit the community. Mr. Kaew, a 72-year-old participant, noted, “*A day care center would provide a space for health-related activities and social interaction among the elderly*”.

(3) *Environmental Accommodations*: to create a supportive and inclusive environment, the health center should carry out the following:
  7. Conduct home safety assessments for elderly participants to identify potential hazards and recommend modifications.
  8. Integrate age-friendly design elements, such as ramps, handrails, and adjustable seating, to enhance mobility and comfort.

By addressing these facility, activity, and environmental requirements, daycare centers can become vital hubs for elderly care, fostering a sense of belonging and community while promoting the overall quality of life for their participants. This holistic approach ensures that the needs of the elderly are met in a dignified, respectful, and sustainable manner.

#### 3.1.6. Living Conditions of the Elderly Participants

(1) *Quantitative Assessment*

We conducted a quantitative assessment of the elderly participants’ living arrangements, summarized as follows:

1. *Living Alone*: approximately 7.1% (15 individuals) of elderly participants live alone without residing with any family members or caregivers.
2. *Living with Spouse Only*: a significant proportion (30%, 64 individuals) resides solely with their spouse, without the presence of children or extended family members.
3. *Living with Children or Grandchildren*: over a third of the participants (34.3%, 72 individuals) live in multigenerational households with their children or grandchildren, reflecting traditional Thai family structures.
4. *Living with Spouse and Children or Grandchildren*: approximately 27.1% (57 individuals) reside with both spouse and children or grandchildren, indicating prevalent multi-generational living arrangements.
5. *Living with Spouse and Extended Family*: a small proportion (0.5%, 1 individual) live with their spouse along with extended family members.
6. *Living with Children or Grandchildren and Extended Family*: approximately 0.5% (1 individual) reside with their children or grandchildren and extended family members.

These statistics highlight diverse living contexts among elderly participants, with significant proportions residing in multi-generational households, while others live in arrangements that may increase their vulnerability to social isolation.

(2) *Qualitative Insights*

Qualitative insights from semi-structured interviews added valuable context to these quantitative findings:

- Participants who live alone often reported concerns about safety, difficulties accessing healthcare, and feelings of loneliness. As one 72-year-old participant stated:
- “I live alone because my children work in another province. They visit sometimes, but I feel lonely most days”.
- Elderly participants living with their children or grandchildren frequently served in caregiving roles, actively contributing to their family’s daily routines. Another participant shared:
- “Even though I’m old, I still help take care of my grandchildren while their parents work”.
- Experiences of elderly individuals in extended family households varied significantly, with some reporting robust familial support while others described feelings of neglect and insufficient attention within their larger family settings.
(3) *Implications for Elderly Care*

These findings emphasize the need for targeted interventions:

- Elderly individuals living alone require community-based support such as regular health checks and social engagement programs to mitigate isolation and improve safety.
- Families caring for elderly members require additional resources and education to better manage caregiving responsibilities and reduce associated stress. Multi-generational family arrangements should be carefully evaluated to ensure adequate support for elderly individuals experiencing neglect or isolation despite living with extended family.

### 3.2. Phase 2: Development of a Daycare Center for the Elderly with Community Participation

The development of the daycare center for the elderly was achieved through a participatory approach, involving four key steps that ensured alignment with community needs and the effective engagement of stakeholders:

***Step 1: Planning the Operations of the Daycare Center:*** Planning began with collaborative discussions to identify the appropriate site, participants, and services. Key components included the following:

- *Location Determination*: A community meeting decided to utilize the multipurpose building in Baan Kho Community, Village 15, Baan Kho Subdistrict, Phon Sawan District, Nakhon Phanom Province, as the daycare center’s location. This site was selected due to its accessibility and central location within the community.
- *Establishment of a Working Group*: A multidisciplinary working group was formed to oversee the center’s operations. This group included representatives from key community stakeholders: The president of the elderly club, representatives from the local health promotion hospital (RPH), officers from the Subdistrict Administrative Organization (SAO), the village headman, and community health volunteers.
- *Target Group Identification*: The working group identified elderly individuals in need of services at the daycare center. Selection criteria included health status, socioeconomic conditions, and potential benefits from participation.
- *Service Design*: The center’s services were scheduled to operate from Monday to Friday, with health rehabilitation services provided once a week. The comprehensive service packages included the following:
  (1) *Health Services*: regular health screenings, nutritional guidance, cooking demonstrations, shared meals, health rehabilitation sessions, brain exercises (e.g., making sandalwood flowers and traditional crafts), and access to traditional medicine.
  (2) *Social Services:* activities such as folk music, music therapy, organic gardening, family support for household vegetable gardens, legal advice, and haircut services.
  (3) *Psychological Services*: listening to Buddhist teachings, relationship-building activities with grandchildren, and participating in community religious events and festivals.
  (4) *Economic Services*: vocational training, market linkage for product sales, selling community-made products, and establishing a savings fund.
  (5) *Environmental Services*: conducting International Classification of Functioning, Disability, and Health (ICF) surveys for home modifications and designing adjustments to suit the elderly’s specific needs.

By systematically addressing these aspects, the planning phase ensured that the daycare center was designed to comprehensively meet the diverse needs of the elderly, leveraging community resources and fostering a sense of collective ownership. This participatory approach not only promoted sustainability but also strengthened community bonds, making the daycare center a valuable asset for elderly care.

***Step 2: Implementation***

The operational phase focused on delivering the planned services in collaboration with researchers and community members. This collaboration ensured that all stakeholders were familiar with the operational plan and were actively involved in service provision. The implementation included the following:

- Coordinating activities according to the schedule.
- Engaging key community leaders and volunteers to ensure seamless execution.
- Establishing communication channels to address immediate concerns during service delivery.

***Step 3: Observation***

This step involved systematically monitoring and evaluating the execution of activities to ensure alignment with the planned objectives and to capture real-time feedback.

- *Monitoring Operations*: Researchers and stakeholders observed the execution of services to verify that activities were conducted as planned. This included ensuring adherence to schedules, availability of resources, and the effectiveness of service delivery.
- *Behavioral Observation*: The behavior and engagement levels of elderly participants were closely monitored. This helped identify patterns of participation, preferences, and any challenges faced by the elderly during activities.

***Step 4: Reflection***

Reflection was a critical step for synthesizing observations and improving future implementation phases. This included the following:

- *Data Analysis*: Researchers analyzed qualitative and quantitative data collected during the implementation and observation phases. This analysis identified key issues, barriers, and areas requiring improvement.
- *Group Meetings*: regular meetings were held with activity organizers and stakeholders to discuss findings, share experiences, and collaboratively explore solutions to identified challenges.
- *Plan Adjustment*: Feedback from data analysis and group meetings was used to refine the operational plan. Adjustments were made to improve service delivery and participant satisfaction.

Researchers conducted two rounds of action research, incorporating lessons learned from each phase, before finalizing the complete set of services.

### 3.3. Phase 3: Evaluation of Daycare Services for the Elderly

The evaluation phase focused on assessing the effectiveness of the services provided by the daycare center for the elderly. This phase included a comprehensive review of operations and outcomes, covering four key aspects to ensure that the center met its objectives and addressed the needs of the elderly.

- *Evaluation Process through Lesson Summarization*

A workshop was conducted to summarize lessons learned from the operations of the daycare center. This activity involved key leaders who participated in the center’s service delivery, providing insights into its development and impact. The key lessons included:
  (1) *Development Stages of the Baan Kho Elderly Daycare Center:* the daycare center originated from the Baan Kho Elderly Club, which had previously collaborated with the Node Health Promotion Foundation due to its exemplary work in elderly care.
    ○ January 2021: initial activities were held on temple grounds, which served as the first venue.
    ○ February 2021: activities were relocated to the SME Pavilion in the village, a space better suited to the nature of the center’s operations and the elderly’s needs.
  (2) *Goals for Establishing the Daycare Center*: the primary aim was to create a central hub where elderly individuals could gather and participate in a variety of activities tailored to their needs.
    ○ Activities were designed to be voluntary, ensuring that participation was based on interest and willingness.
    ○ Retired professionals were encouraged to volunteer, contributing their expertise to the center’s programs.
    ○ The center sought collaboration with external organizations for knowledge sharing and resource donations, enhancing its operational capacity.
  (3) *Community Expectations for the Daycare Center:* the community envisioned the center as a venue that would accomplish the following:
    ○ Provide activities tailored to the elderly’s physical, mental, and social needs.
    ○ Promote cheerfulness and stress relief among the elderly.
    ○ Serve as a daily activity center equipped with adequate facilities to support elderly engagement.
  (4) *Development Path of the Elderly Daycare Center*: the step-by-step progress and milestones of establishing the center were documented in detail, highlighting the systematic approach taken to create and enhance the center’s operations.

II. *Meeting the Needs of the Elderly*
  The evaluation ensured that the daycare center’s services aligned with the specific requirements of the elderly. This included providing health, social, psychological, and vocational support while addressing environmental needs.
III. *Providing Community Value*
  The center was assessed for its contribution to the community, particularly in fostering social cohesion, reducing caregiver burdens, and involving community members and organizations in elderly care.

**Figure 4:**
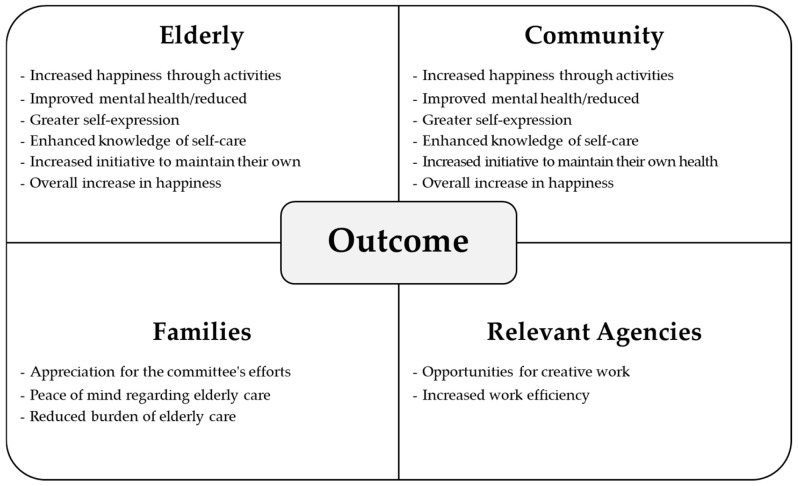
Outcomes of the Operations of the Elderly Daycare Center.

IV. *Achieving Intended Goals*
  The evaluation verified that the center achieved its goals, including creating a welcoming and functional hub for the elderly, promoting well-being, and establishing a sustainable model for elderly care.
  By evaluating these aspects, the research provided evidence of the center’s success in enhancing the quality of life for the elderly while meeting the community’s expectations. The findings demonstrated the effectiveness of community-driven initiatives in establishing sustainable and impactful care models (See Figure 4).

***Positive Impacts and Outcomes of the Elderly Daycare Center in Baan Kho Community***

The daycare center’s operations demonstrated significant benefits for various stakeholders, including the elderly, the community, families, and relevant agencies. The findings are summarized under two key areas:

I. ***Lessons Learned from Developing the Daycare Center***

The development process revealed key mechanisms and support structures crucial to the center’s success:
  (1) *Main Mechanisms:* four primary components were instrumental in establishing and sustaining the daycare center:
    1. *Elderly Club*: acted as the core group for coordinating activities and promoting participation.
    2. *Subdistrict Health Promotion Hospital (RPH)*: provided health services and expertise.
    3. *Volunteer Teachers/Artisans*: facilitated skill-building and vocational activities.
    4. *Village Health Volunteers (VHVs)*: supported health-related initiatives and outreach efforts.
  (2) *Supporting Mechanisms*: A wide range of stakeholders contributed to the center’s development and operations, including the following:
    1. Academic institutions and hospitals for technical and financial support.
    2. Temples for spiritual and cultural activities.
    3. Village headmen and Subdistrict Administrative Organizations (SAOs) for administrative and logistical support.
    4. Non-formal education centers and foundations for additional resources and training.

II. ***Outcomes of the Elderly Daycare Center***

The elderly daycare center in Baan Kho Community marks a key achievement in improving the quality of life for elderly individuals. Using a community-driven approach, the center integrates health, social, vocational, psychological, and environmental services to address diverse needs. It fosters active participation and utilizes local resources, benefiting not only the elderly but also families, the community, and related agencies. Systematic evaluations, including satisfaction surveys and comparative analyses, highlight its effectiveness in promoting well-being and generating innovations. These outcomes showcase the center’s value as a model for addressing aging challenges through community-based care.
  (1) *Creation of New Knowledge and Innovations*: The establishment of the center led to the development of innovative service packages tailored to the needs of the elderly. These included diverse daily activities that promoted physical, mental, and social well-being, such as health rehabilitation, traditional crafts, and cultural events.
  (2) *Improvement in Well-being and Satisfaction*: The effectiveness of the center was evaluated through a comparative analysis of data collected before and after elderly participation in its activities. Satisfaction surveys from 50 elderly respondents were analyzed using the *t*-test-dependent statistical method, revealing significant improvements in their well-being and quality of life (See Table 6). The analysis highlighted (1) increased satisfaction with the center’s services, (2) enhanced physical and mental health outcomes, and (3) greater social engagement and reduced feelings of isolation. These findings underscore the critical role of community-driven initiatives in improving elderly care. The lessons learned and positive outcomes demonstrate the potential for replicating this model in other communities to address the comprehensive needs of elderly populations.

**Table 6:** Analysis Results of the Satisfaction Survey before and after attending the Daycare Center.

Satisfaction with Services at Baan Kho Elderly Daycare Center	*n*	Mean	SD	Mean Difference	SE	95% Mean Difference		T	df	*p*-Value
						Lower	Upper			
After the test	51	3.57	0.35	−0.59	0.06	−0.71	−0.47	−9.91	50	0.000
Before the test	51	4.16	0.29	−0.59	0.06	−0.71	−0.47	−9.91	50	0.000

Table 7 shows that the comparison of health data before and after participating in activities revealed statistically significant changes among the elderly. The weight of the elderly decreased significantly after participating in the activities (mean difference = 0.64, sig. 0.00). The body mass index (BMI) also decreased significantly (mean difference = 0.28, sig. 0.00). The ability to perform daily living activities, measured by the Barthel ADL index, increased significantly (mean difference = −0.20, sig. 0.05). Additionally, the scores from the dementia assessment test increased significantly (mean difference = −4.66, sig. 0.00).

## 4. Discussion

The findings from the three phases of this research provide a comprehensive understanding of the development, implementation, and evaluation of a daycare center for the elderly in the Baan Kho community. These results highlight the critical role of community participation and the multifaceted impacts on the elderly and other stakeholders involved.

### 4.1. Lessons Learned from Developing the Daycare Center

The development of the Baan Kho Elderly Daycare Center was primarily driven by the involvement of key community components: the Elderly Club, the Subdistrict Health Promotion Hospital (RPH), volunteer teachers and artisans, and village health volunteers (VHVs). Supporting mechanisms included academic institutions, hospitals, temples, village headmen, and Subdistrict Administrative Organizations (SAOs). This multi-stakeholder approach was crucial in ensuring a sustainable and effective development process. The importance of such community-driven initiatives has been emphasized in various studies, which show that engaging local resources and stakeholders leads to more culturally relevant and sustainable care models [5,8]. The initial phase of the study revealed significant socioeconomic challenges faced by the elderly, including chronic health conditions, low income levels, and limited access to adequate care. These findings underscore the need for comprehensive support systems that address not only health needs but also social, economic, and environmental factors [2,4]. A key lesson learned is the importance of a realistic cost assessment for policymakers, local governments, and stakeholders, ensuring that such models can be replicated and adapted based on available resources.

(1) *The Role of Realistic Cost Assessment in Policy Planning:* For policymakers and stakeholders, understanding the financial requirements of an elderly daycare center is critical in determining its feasibility and long-term sustainability. Studies have shown that community-based elder care models can be more cost-effective than institutional care if resources are utilized efficiently. Our study demonstrated that high-quality elderly care services can be provided at a relatively low cost, particularly when leveraging community participation and existing public health infrastructure [8].
(2) *Balancing Cost and Service Quality:* One challenge in elderly care services is maintaining affordability without compromising quality. A systematic review by Orellana, Manthorpe, and Tinker [9] found that daycare centers significantly improve elderly well-being and reduce healthcare costs by preventing hospitalizations and long-term institutional care. The Ban Kho model achieved this balance by utilizing existing community infrastructure instead of investing in costly new facilities [13]; establishing partnerships with health promotion hospitals, which provided free health check-ups and medical supervision [14]; engaging retired professionals and trained volunteers, which eliminated the need for expensive full-time staff [15]; and offering flexible service packages, allowing local governments to scale services up or down depending on their financial capacity [16]. These approaches ensured that elderly individuals received essential care, social interaction, and health services without imposing unsustainable financial burdens on local governments [17].
(3) *The Importance of Public-Private Collaboration:* The study highlights that a multi-sectoral funding approach is necessary for financially sustainable elderly care centers [2]. The project was able to maintain operations without charging fees to elderly participants due to diversified funding streams, including public sector investment (local government and public health agencies) [18], private donations (community fundraising, local businesses, and religious organizations) [8], academic grants, supporting training programs and caregiver education [19], and volunteer support, reducing labor costs [13].

For policymakers, this model suggests that public–private partnerships (PPP) can enhance service delivery efficiency while ensuring long-term financial sustainability [6].

(4) *Cost-Effectiveness and Scalability for Broader Policy Implementation:* For policymakers and stakeholders looking to expand elderly daycare services, the Ban Kho model provides an adaptable framework that can be implemented in other regions [9]. Given that Thailand’s aging population is growing, a cost-effective and scalable model for elderly care must be integrated into national and local policy frameworks [20]. The Ban Kho model’s ability to operate at ~12,416 USD/year PPP-adjusted demonstrates that sustainable elderly care is financially achievable, even in low-resource settings [8,21].

### 4.2. Outcomes of the Daycare Center

The establishment of the daycare center demonstrated meaningful improvements in the health and well-being of elderly participants. The center’s comprehensive service package, which included health screenings, nutritional guidance, physical and cognitive activities, and social engagement, played a pivotal role in enhancing participants’ overall quality of life. These services addressed critical areas such as chronic disease management, mental stimulation, and opportunities for social interaction, reflecting the center’s holistic approach to elderly care. The improvements observed in this study align with previous research emphasizing the effectiveness of structured, community-based programs in enhancing physical and mental health outcomes among elderly populations. Community-driven initiatives, such as the daycare center in Baan Kho, provide a sustainable model for addressing the multidimensional needs of aging populations through integrated care services [8]. By fostering active participation and utilizing local resources, the center not only supported the elderly in maintaining their independence but also contributed to broader social cohesion and community resilience [7].

The findings of this study further underscore the value of community-based interventions in improving elderly care outcomes. Future studies should investigate the scalability and adaptability of such models across different demographic and cultural contexts to address the growing challenges of global aging [9,13].

### 4.3. Impact on the Community and Stakeholders

The daycare center not only provided significant benefits to the elderly but also positively impacted their families and the broader community. The increased awareness of the center’s services fostered stronger community ties and enhanced coordination between the elderly and other community members. Families expressed gratitude for the center, highlighting how it provided peace of mind and alleviated the caregiving burden. These findings align with existing research that emphasizes the role of community centers in fostering social cohesion and offering vital support to families [5]. The project also benefited relevant agencies involved in the initiative. Organizations such as health promotion hospitals, subdistrict administrative organizations, and local foundations experienced increased opportunities for creative collaboration and improved work efficiency. These collaborative efforts demonstrated the broad-reaching advantages of the daycare center model, which not only addressed the complex needs of the elderly but also strengthened the community’s capacity to support its aging population [8].

The integration of various stakeholders into the planning and operational processes underscored the importance of a coordinated approach to elderly care. By leveraging local resources, fostering collaboration, and promoting community involvement, the daycare center created a comprehensive support system that addressed the multifaceted challenges of aging. These outcomes highlight the potential of community-based models to enhance social cohesion, reduce familial burdens, and improve service delivery efficiency across diverse contexts. Future efforts should focus on scaling and adapting such initiatives to meet the needs of aging populations in other regions.

### 4.4. Impact on Geriatric Health

The study demonstrated improvements in well-being, social participation, and cognitive function. To provide a broader context, we compared our findings with previous daycare center models in other countries and urban settings. Studies in Japan, Sweden, and the United States have reported similar benefits in cognitive stimulation and social engagement among elderly daycare participants. However, unique to our study is the strong role of community participation, which may contribute to higher adherence and cultural acceptability in rural settings. While the study primarily focused on general well-being, further analysis has been added regarding key geriatric health indicators. Although frailty and sarcopenia were not initially assessed, indicators related to these conditions—such as grip strength and mobility—can be integrated into future evaluations. Additionally, the program’s impact on fall prevention was considered through balance-enhancing activities, though specific fall rates were not tracked in this study. Future iterations of the model should incorporate validated frailty and sarcopenia assessments to strengthen clinical relevance.

*Model Sustainability:*

The study mentions scalability to other communities but now includes a cost-effectiveness analysis. The estimated annual operating cost of the daycare center was compared to institutional elderly care costs, demonstrating that community-based models are financially more sustainable while maintaining service quality. To enhance long-term financial sustainability, a multi-funding strategy is proposed:

(1) Government Funding: ongoing financial support from local administrative organizations and public health programs.
(2) Community Contributions: a combination of local fundraising, voluntary donations, and Corporate Social Responsibility (CSR) initiatives.
(3) Public–Private Partnerships: collaboration with private healthcare providers and non-profit organizations to co-fund operational expenses.

Microfinance and Cooperative Models: encouraging elderly participants to engage in self-sustaining activities, such as community-based enterprises, that contribute to daycare center funding.

## 5. Conclusions

This research successfully developed a sustainable and effective daycare center service model for the elderly through a participatory approach in the Baan Kho community. Utilizing participatory action research methods, the study addressed the multifaceted needs of the elderly, encompassing health, social, economic, and environmental dimensions. The active involvement of key community stakeholders, including the Elderly Club, Subdistrict Health Promotion Hospital, volunteer teachers and artisans, and village health volunteers, was instrumental in ensuring the project’s success. The daycare center significantly enhanced the well-being and quality of life of elderly participants. Statistically significant improvements in physical health indicators, such as weight and BMI, daily living activities, and cognitive function (dementia test scores), demonstrate the effectiveness of the comprehensive service package provided. Beyond individual benefits, the center positively impacted the wider community by fostering stronger social connections, alleviating the caregiving burden on families, and improving work efficiency for relevant agencies.

This model offers a replicable framework for enhancing elderly care in semi-urban and semi-rural contexts. Its community-driven approach not only addresses the immediate needs of the elderly but also fosters a supportive and engaged community, ensuring the cultural relevance and sustainability of care services. The findings contribute to the growing body of evidence supporting community-based care models and emphasize the critical role of local participation in the design and implementation of elderly care services. This study highlights the potential for similar initiatives to be adapted and scaled in diverse settings to address the challenges of an aging population globally.

## Figures and Tables

**Table 1:** The estimated costs of setting up and running the daycare center.

Cost Category	Estimated Amount (THB)	PPP-Adjusted (USD)	Funding Source
Infrastructure Setup and Renovation	150,000	~9804	Local Government and Community Fundraising
Equipment and Materials (furniture, kitchen, recreational materials)	50,000	~3268	Local Government and Donations
Health Services (screenings, basic medical supplies)	30,000/year	~1961/year	Public Health Center and Volunteers
Daily Operations and Staff Support	100,000/year	~6536/year	Local Administrative Organization (SAO)
Community and Educational Activities (workshops, training, recreational programs)	40,000/year	~2614/year	Public and Private Donations
Volunteer and Support Incentives	20,000/year	~1307/year	Local Health and Social Welfare Budget
Miscellaneous Expenses (transportation, maintenance)	20,000 B/year	~1307/year	Local Government

**Table 2:** The socioeconomic information of the elderly.

Characteristics	*N* (%)
Male	71 (33.81)
Female	139 (66.19)
Age	
Young old (60–69 years old)	304 (56.93)
Middle old (70–79 years old)	175 (32.77)
Very old (80 years old and above)	54 (10.11)
Nonelderly (Under 60 years of age)	1 (0.19)
Marital status	
Single	7 (3.33)
Marriage	119 (56.67)
Widowed	78 (37.14)
Divorce	6 (2.86)
Education	
Bachelor’s degree	1 (0.48)
Secondary education	4 (1.90)
Primary education	195 (92.86)
Not educate	10 (4.76)
Occupation	
Farmer	153 (72.86)
Owner business	14 (6.67)
Unemployed	39 (18.57)
Not specified	4 (1.90)

**Table 3:** Monthly Income Distribution of Elderly Participants: THB and Equivalent PPP USD Compared to Global Standards.

Income per Month (THB)	Equivalent in PPP USD	Comparison with Global Standards	*N* (%)
Less than 1000	~65	Below extreme poverty line (USD 2.15/day)	130 (61.90)
1001–3000	65–196	Near/below lower-middle-income threshold	71 (33.81)
3100–5000	~203–327	Low income, but above extreme poverty	6 (2.86)
5001–10,000	~327–654	Comparable to low–middle income	2 (0.95)
More than 10,000	>654	Closer to global lower-middle income	1 (0.48)

**Table 4:** Health Problems of the Elderly and the Health Needs of the Elderly.

Issues	Health Problem		Health Needs of the Elderly	
	Mean (SD)	Interpretation	Mean (SD)	Interpretation
Physical	1.61 (0.85)	Very Low	4.33 (0.90)	High
Mental	1.57 (0.76)	Very Low	4.21 (0.90)	High
Social and Economic	2.77 (1.49)	Low	4.27 (0.94)	High
Spiritual	1.67 (0.84)	Very Low	-	-
Housing and Environment	1.32 (0.60)	Very Low	4.18 (0.89)	High
Overall Questionnaire	1.79 (0.90)	Very Low	4.25 (0.91)	High

**Table 5:** Survey Results on the Needs for Daycare Services for the Elderly.

Daycare Service Needs	Mean	Standard Deviation	Interpretation
Need for Daycare Center Establishment	4.47	0.77	High
Service Needs	4.33	0.82	High
Facility Needs	4.20	0.87	High
Overall Questionnaire	4.33	0.82	High

**Table 7:** The comparison of health data before and after participating in activities.

Health Data Before and After	Mean Difference	S.D.	*t*-Value	df	Sig.
Weight	0.64	1.18	3.83	49	0.00
Body Mass Index (BMI)	0.28	0.50	3.94	49	0.00
Ability to perform daily living activities (Barthel ADL index)	−0.20	0.61	−2.33	49	0.02
Dementia status	−4.66	2.98	−11.06	49	0.00

## Data Availability

The data supporting the reported results of this study are available upon request from the corresponding author. Due to privacy and ethical restrictions, the data are not publicly available. Access may be granted for research purposes following approval from the Institutional Review Board of Nakhon Phanom University and subject to applicable data-sharing agreements.
